# Keeping the conversation going: How progressivity is prioritised in co-remembering talk between couples impacted by dementia

**DOI:** 10.1177/13634593221127822

**Published:** 2022-10-13

**Authors:** Felicity Slocombe, Elizabeth Peel, Alison Pilnick, Saul Albert

**Affiliations:** Loughborough University, UK; Loughborough University, UK; University of Nottingham, UK; Loughborough University, UK

**Keywords:** Conversation analysis, co-remembering, dementia, progressivity, relationality

## Abstract

This article explores how partners keep the conversation going with people living with dementia (PLWD) when speaking about shared memories. Remembering is important for PLWD and their families. Indeed, memory loss is often equated with identity loss. In conversation, references to shared past events (co-rememberings) can occasion interactional trouble if memories cannot be mutually recalled. This article analyses partners’ interactional practices that enable progressivity in conversations about shared memories with a PLWD. In previous research, both informal and formal carers have reported that they can find interacting with PLWD difficult. Identifying practices used by partners is one way to begin addressing those difficulties. Analytical findings are based on over 26 hours of video data from domestic settings where partners have recorded their interactions with their spouse/close friend who is living with dementia. The focus is on 14 sequences of conversation about shared memories. We show how particular practices (candidate answers, tag questions and single-party memory of a shared event) structure the interaction to facilitate conversational progression. When partners facilitate conversational progressivity, PLWD are less likely to experience stalls in conversation. Our findings suggest the actual recall of memory is less relevant than the sense of shared connection resulting from the conversational activity of co-remembering, aiding maintenance of individual and shared identities. These findings have relevance for wider care settings.

## Introduction

Social interaction involves co-participation. When one person interacts with another, they are part of a shared experience (Rae and Ramey, 2020). Co-remembering or shared remembering is a specific form of co-participation. Dementia poses challenges to interactional co-participation through its impact on relationships and communication. Dementia can lead to changes in roles and power, which can threaten individual identity, as well as relationships (Wadham et al., 2016). As dementia progresses it can also cause changes in communicative abilities for PLWD (Banovic et al., 2018; Nyström and Lauritzen, 2005) and create problems in communicating effectively with people living with dementia (PLWD) for practitioners (Young et al., 2011), care staff (O’Brien et al., 2020) and family members (Savundranayagam and Orange, 2014). Interactions with others can help maintain the identity of PLWD (Cowdell, 2006; Kitwood, 1997). Specifically, storytelling about past shared events allows people to convey belonging and identity (Hydén, 2011, 2017). A meta-analysis of randomised controlled trials found speaking about the past has therapeutic benefits for PLWD (Park et al., 2019). It may be that partners can replicate some of these benefits (including reduction in depression and other behavioural and psychological symptoms) through co-remembering in the home environment. There has been a call for more research into the approaches used by conversation partners to support co-remembering with PLWD as few studies have focused on this (Hydén, 2011). This is grounded in a need to support communication for PLWD and their partners to maintain individual and shared identities.

Remembering is also a deeply social activity (Middleton and Edwards, 1990). When memories are drawn upon conversationally, they are often tailored to the topic of the ongoing conversation (Sacks, 1992). For example, if talking about a future holiday, a person may recall an event from a previous holiday. Conversations about shared memories can link to identity, through performing a co-construction of self (Crichton and Koch, 2011) in talking about something all parties have experienced. This is especially significant for couples where one person is diagnosed with dementia, as there may be problems with accessing memories and life stories (Williams et al., 2019). Theories of relationality are useful for exploring co-remembering and dementia in the setting of close domestic relationships. In this article close domestic relationships include both romantic and platonic dyads. The term relationality refers to how everyone is rooted in and supported through our relationships (Peel and Harding, 2015). We do not exist in an individual vacuum, we exist in a relational context (Harding, 2017). Indeed, some of our most fundamental characteristics (e.g. our capacity for language) are developed through our interactions with others (Nedelsky, 1989). Dimensions of ourselves are linked to our relationships with others, and it is important that the relationships valued by PLWD are recognised (Harding, 2017). One way we can see relationality in interactions is through co-remembering. In this situation, discussing shared memories can lead to an increase in shared positive attitude, helping both people to appreciate the present moment (Molyneaux et al., 2012; Robinson et al., 2005). By contrast, when PLWD are unable to share memories and stories, research shows that this is experienced as distressing for family members (Small et al., 2003). The interactional practices situated within co-rememberings have been little investigated in the field of conversation analysis (CA) (but see Bolden and Mandelbaum, 2017; Williams et al., 2019). Bolden and Mandelbaum (2017) explored how co-rememberings are used interactionally as evidence to support a contentious claim outside of a dementia context, and Williams et al. (2019) showed how knowledge relating to a PLWD’s memories is constructed by other interlocuters who use co-rememberings in their talk. Williams et al. (2019) analysed strategies for reducing possible interactional trouble in this context. However, the setting of their research was dementia groups where reminiscence and talking about the past were pre-identified topics for discussion. In this article, the setting is PLWD and their partners in domestic contexts and where co-remembering has arisen from mundane conversation.

Additional previous research has investigated ‘we-ness’ in interaction, and this can be used to explore the co-element of co-remembering. In relationships between close individuals, the use of ‘we’ in talk can function to maintain and (re)construct the couple relationship (Hydén and Nilsson, 2015). Hydén and Nilsson (2015) examined pronoun use (specifically ‘we’) and how it positions couples (where one is living with dementia) in relation to one another. Following in similar methodological steps to Hydén and Nilsson (2015), we use CA to interrogate interactions in this article. CA is employed to examine practices for maintaining progressivity by partners when engaged in sequences of co-remembering with a PLWD. CA asks, ‘why this now?’ (Schegloff and Sacks, 1973: 299) and focuses on how each turn of talk impacts the sequence of the interaction, centering the *action* in interaction (e.g. Ten Have, 2007). In CA research it is common for analysis and findings to be based upon what may appear a small sample, or even one sequence of interaction (known as a single case analysis; Schegloff, 1987, 1993). In this article, 14 sequences of interaction are drawn upon. This type of analysis is often used when research surrounding the phenomena of interest already exists – in this instance research on tag questions, candidates, co-remembering and storytelling. As Schegloff (1987) highlighted with respect to the scope and focus of CA analyses: ‘the resources of past work on a *range of phenomena* and organizational domains in talk-in-interaction are brought to bear on the analytic explication of a *single fragment* of talk’ (p. 101, original emphasis). Although the present research is not a single case analysis, there are relatively few sequences. In CA research, and the present article, this type of analysis can be used to apply existing research findings on the phenomena to a new interactional setting (Schegloff, 1987). Several CA related principles require foregrounding for our analysis: structural preference; the cross-cutting preferences of progressivity and intersubjectivity; repair; co-tellings and recipient design.

Structural preference relates to the way an interlocuter designs their talk and responds to the talk of others. For example, the structurally preferred response to an invitation is an acceptance rather than a rejection (Pomerantz and Heritage, 2012). The interactional principles of intersubjectivity and progressivity have cross-cutting preferences. Intersubjectivity is the joint or shared understanding between persons (Sidnell, 2010), while progressivity is about ‘moving from some element to a hearably-next one with nothing intervening’ (Schegloff, 2007: 15). Schegloff (2007) describes how the structurally preferred response to an interlocuter’s first turn of talk in a sequence is a response which furthers or completes the ongoing activity (e.g. accepting the invitation, agreeing with an assessment, offering an apology to a complaint). When asking a previous speaker to clarify what they have said, intersubjectivity is being preferred over progressivity by the recipient – they have stopped the ongoing conversation so that something is clarified (Heritage, 2007). Asking the previous speaker to clarify their talk is also one way of doing repair.

Repair occurs when an interlocuter addresses an utterance which has caused an interactional problem in hearing, understanding or speaking (Schegloff et al., 1977). This could be a recipient saying ‘huh?’ or ‘what?’ (other-initiated repair; Drew, 1997), or a speaker changing a word part way through speaking it, such as ‘it was small- larger’ (self-initiated self-repair; see Schegloff et al. (1977) for discussion of repair). By initiating repair, a person opens themselves up to being heard as interrupting the progressivity of the ongoing interaction (Schegloff, 2007), but repair is also the resource through which intersubjectivity is secured (Heritage, 2007). Many practices can contribute to progressivity, some of which will be the focus of this article. Previous CA research has identified that in talk which favours progressivity, the nature of intersubjectivity is inferred (Heritage, 2007). Interlocuters work together to keep the conversation going, even in a situation where a question has been asked to someone who is having difficulty providing an answer, as others will work to promote progressivity (Goodwin and Goodwin, 1986; Stivers and Robinson, 2006). Previous research has examined how PLWD’s use of repair changes throughout the progression of the condition. In early-stage dementia, requests by PLWD for others to repair their talk happen more, but the percentage of conversation involved in repair is significantly higher for middle-stage dementia (Orange et al., 1996). Other research has interrogated practices used for repairing/correcting the talk of PLWD (Landmark et al., 2021). Landmark et al. (2021) found that in the couple relationships present in their participants, there were more allowances for the partner to correct the PLWD on a topic or issue which may mainly be in the knowledge domain of the PLWD. This can be seen as ‘taking a minor liberty’ relative to the ordinary expectations of interaction (Landmark et al., 2021) but can also be seen as displaying the couple relationship by the partner knowing information about the PLWD (Goffman, 1971). There is also work analysing the ways in which healthcare professionals maintain progressivity with PLWD in the acute hospital setting (Pilnick et al., 2021). This research found that in response to hard-to-interpret talk from PLWD, healthcare professionals use a range of practices, such as responding to the emotional tone of the PLWD’s talk, or shifting to a different topic, in order to avoid repair (Pilnick et al., 2021). In this article, we similarly analyse practices to promote progressivity used in conversation with PLWD, but in the non-institutional context of the home and with partners of PLWD, rather than healthcare professionals.

Co-rememberings are examples of storytellings, or tellings, a topic which there has a long tradition in CA research (Goodwin, 1984; Kasper and Prior, 2015). CA research has shown how storytellings, or tellings are jointly accomplished by tellers and recipients (Sacks, 1992); they are achieved in collaboration with others, like co-rememberings. Co-tellings (Hayashi et al., 2002) or collaborative storytellings (Kindell et al., 2017) are the terms used within the CA literature to refer to events or stories which are reported on collaboratively in conversation. Conversation analytic work has shown the systematic nature of how storytellings unfold: how a teller negotiates their authority to tell a story and how they may preface the story by requesting/offering to tell a story (Hayashi et al., 2002; Sacks, 1992). Additionally, it is important for a teller to indicate how the story relates to the ongoing topic of conversation (Jefferson, 1978). Also, important to consider is who has the rights and obligations to participate in a telling. This is known as epistemics: the right to claim, assert, contest or defend knowledge (Heritage, 2012, 2013). There is a growing body of CA work on epistemics-in-interaction, though there is not space to consider this in detail here. However, as Landmark et al.’s (2021) work suggests, interactions with PLWD may disrupt some standard epistemic assumptions.

Lastly, recipient design refers to how a speaker puts together the words they use in a way that is designed for the recipient of the talk (Sacks et al., 1974). Recipient design encompasses many of the CA principles already discussed. For example, co-tellings are designed to be on the topic of something which both the recipient and speaker have experienced. Indeed, designing talk for progressivity involves designing talk in a way which structurally prefers for the recipient to progress the interaction (e.g. through agreement). Recipient design is about how a speaker designs their talk in a ‘context-sensitive’ manner (Sacks et al., 1974: 727) and is an important concept not only to CA but also talk with PLWD (Williams et al., 2019). In this article, we aim to demonstrate how partners of PLWD design their talk to fit the context of interacting with their loved one living with dementia.

Previous research has focused on how others can support PLWD to retrieve memories (e.g. Kemper et al., 1995), but in this article we aim to analyse how partners design their talk so that it is unnecessary for the PLWD to display retrieval of memory. In what follows, we will argue that by designing talk for progressivity, participants can reduce the stakes for maintaining intersubjectivity, reduce opportunities for the surfacing of interactional trouble, and minimise the need for repair. In doing this, partners support identity maintenance for the PLWD as well as relational shared identity. This article contributes to the call for more research on how conversation partners support PLWD in co-remembering (Hydén, 2011).

## Method

The data was collected from March to July 2012 as part of the Dementia Talking project (e.g. Peel 2014, 2015). The Social Care REC approved the study. This article draws upon 26.5 hours of video data, none of which has previously featured in publications. These data feature three dyads, where one member of each dyad is clinically diagnosed with dementia. The participants were recruited from having taken part in a previous study; 3 out of 16 eligible informal care partners agreed to participate (response rate = 16.6%). Those who did not participate gave varying reasons: increase in severity of PLWD’s condition, PLWD had moved into residential care, or death of PLWD. Video data was recorded in participant’s homes by participants. Before commencement of the study, the researcher (EP) met with the participants to supply recording equipment (a Kodak PlaySport video camera, tripod and remote) and explained how to use it. EP emphasised an interest in recording ordinary communication (e.g. during mealtimes, when playing games or watching television). When EP collected the equipment (July 2012), a debriefing interview was also undertaken. Participants living with dementia were 2–4 years post-diagnosis at the time of recording.

All participants living with dementia were able to communicate verbally and non-verbally. Every person with a dementia diagnosis is an individual and experiences differing symptoms and levels of these symptoms. Dementia can impact upon language and communication in different ways. In general dementia can affect word processing speed, word-finding, using word substitutes or a related word, the making up of words and difficulty focusing and understanding what was said (Alzheimer’s Society, 2022; Banovic et al., 2018). In the talk of participants in the data, their dementia was apparent in the content of their talk and sometimes other indicators such as processing speed, word-finding and occasional difficulty in providing a relevant response to the previous turn. It is important to note that displayed difficulties are not visible in all data, suggesting that communicative difficulties in PLWD are not static, and alluding to the role of interactional context and communication partners in aiding conversational fluidity (as we hope to demonstrate with our findings). Video data were transcribed using the Jefferson (2004) Transcription System. Please see Jefferson (2004) for explanation of transcription symbols used. In the analysis a line drawing is used to illustrate embodied actions such as gaze and smiling. Table 1 provides demographic information of participants.

**Table 1.** Participant demographic information.

Pseudonyms of dyad (age)	Person living with dementia	Form of dementia diagnosed with (year diagnosed)	Relationship to each other and living arrangements	Ethnicity	Number of hours of video recorded	Number of co-remembering sequences
Graham (87) and June (86)	June	Alzheimer’s disease (2008)	Married – cohabiting	White British	8	4
Claudia (60) and Ewan (53)	Ewan	Original diagnosis of Frontotemporal dementia (2010), subsequently diagnosed with Alzheimer’s disease	Friends – cohabiting	White British	9.5	2
John (72) and Jessica (62)	John	Lewy Body Dementia (2010)	Married – cohabiting	White British	9	8

## Analytical findings

Extracts from the video data are used to illustrate interactional practices for maintaining progressivity employed by partners during co-rememberings with PLWD. The various memories drawn on in conversations could result in interactional problems for PLWD and their partners. If a PLWD does not recall a memory that their partner presents, this presents a challenge to the progressivity of the interaction. Where repair in other contexts may be focused on troubles in speaking, hearing or understanding (Schegloff et al., 1977), in interactions with PLWD, there is the additional problem of remembering.

The interactional practices used by partners of PLWD in this data to maintain progressivity in their co-remembering talk are discussed. The extracts used do not feature Ewan and Claudia as the most explicit examples were used to demonstrate patterns across the 14 sequences of co-remembering. The interactional practices identified were candidate answers, tag questions and single-party memories of a shared event. We discuss how these practices, when used in this context, keep the conversation going with PLWD.

### Candidate answers and tag questions

We first discuss candidate answers and tag questions together as they co-occur very frequently in these data. A candidate answer can be used to guide a co-participant to a suggested answer (Pomerantz, 1988), by proffering a model answer the recipient can choose to accept or reject in their response (Heritage, 1984). Candidate answers can also be used to seek information (Pomerantz, 1988), or check/clarify what a previous speaker meant (Antaki, 2012). Candidate answers can be useful when speaking with those with interactional difficulties as they can direct the recipient towards the sought-after response (Pomerantz, 1988). Tag questions are a type of question that usually feature after a transition relevance place (TRP; Clift, 2016) and have been termed a form of ‘recompleter’ by Sacks et al. (1974) because they function as a device to exit the turn and transfer the conversational floor to another. Tag questions are used when the current speaker has reached a TRP without selecting a next speaker, and/or no other co-participant has self-selected to speak next. Tag questions can therefore be used to facilitate interaction for co-participants who may require support to participate in interaction or otherwise have communicative difficulties, such as PLWD (Kempler, 1991; Welland et al., 2002). Extract 1 below illustrates how candidate answers and tag questions were commonly used by partners of PLWD.

*Extract 1.* Graham and June. 05:31-05:37 into a 15:37 video.

*Graham and June are sat at the kitchen table at their daughter Kate’s house. Kate is stood at the oven preparing their meal and does not contribute verbally to this extract. They have all been discussing the Malvern Flower Show and Graham and June discuss that they have been before and recall whether they had to buy tickets in advance (the co-remembering)*.

1 GRA:   =We’ve been once haven’t we. We went once.

2 JUN:   Yes we did.

3 GRA:   But I can’t remember whether we booked or what.

In Extract 1, Graham brings June into the conversation with his question ‘We’ve been once haven’t we’. In the prior interaction (not shown here), June has been a passive interlocuter and has not said anything beyond laughing just before this extract begins. Graham facilitates June’s involvement in the conversation using the pronoun ‘we’ and the tag question ‘haven’t we’ that selects June as the next speaker and exemplifies their couple relationship through the first-person plural pronoun ‘we’ (Hydén and Nilsson, 2015). When Graham says ‘We went once’ at the end of his turn at line 1, he further facilitates June’s response by providing her with a candidate answer at the point of turn transition (Svennevig, 2012). Graham’s tag question is also positioned to prefer ‘yes’ rather than ‘no’ as the response (Pomerantz and Heritage, 2012). In this instance, a ‘passing yes’ is the preferred response – nothing more than assent is required, reducing the work needed for June to produce a relevant response.

The design of Graham’s turn therefore invites June to participate in the interaction in a way that helps to structure what her response could be. This could limit the responsibility for the kind of answer she produces (Pomerantz, 1988; Svennevig, 2012). The impact of this interactionally for June’s identity is that she can display herself as a skilled interactant who responds to Graham’s turn in a timely manner (there is no pause in between Graham and June’s turn) and her talk at line 2 is treated as relevant (Pomerantz, 1988) as it is not challenged or questioned by Graham or Kate (their daughter who is also present in this interaction). In terms of their couple identity, Graham’s talk can be seen to maintain the relationship himself and June have as a couple together – through use of the ‘we’ pronoun (three times) and through the design of his talk in including June and guiding her answer through use of his candidate answer. Extracts 2 and 3 show further examples of how candidate answers and tag questions can be used to progress the conversation.

*Extract 2*. Graham and June. 02:07-02:15 of 07:56 video.

*Graham and June are sat at the table in their own home. They have just finished playing Boggle (a game where you make words out of random letters). They have recently returned from visiting their other daughter (Polly). Graham has said they are a bit out of practice at Boggle and the extract begins by saying the Boggle box is a bit awkward to transport*.

1 GRA: → It’s a bit bulky to take the (.) Boggle when we go isn’t it,

2 JUN:   [It is really. ]

3 GRA:   [It’s an awkward] box thing really. But.

4      (0.4)

*In between Extract 2 and 3 Graham and June discuss that June learned how to play cribbage (a card game) from her father, which leads into the talk in Extract 3 relating to June’s father*.

*Extract 3*. Graham and June. 03:13-03:30 of 07:56 video.

51 GRA:    Must have been ↑awkward being in the Sunday school class

52    where your Dad was the boss:.

53 JUN:  ((small smile))

54 GRA: heh heh heh heh huh

55     (0.5)

56 GRA: But you didn’t get [any ]

57 JUN:  [>Well] it was only Sunday school.<

58 GRA: → Yeah I know. (.) But you #din’t get any favours# did you.

59 JUN:     ((shakes head))

60 GRA: → £No(h)t really.£

61     (1.1)

62 JUN: → No:, I don’t think so.

In Extract 2 and 3, Graham’s tag questions (line 1 – ‘isn’t it’ and line 58 – ‘did you’) set up a context where there is a preference for June to respond in confirmation, which she does in both instances. The use of tag questions by Graham limits June’s responsibility for the kind of answer she produces and therefore potentially make it easier for her to present herself as a proficient speaker as Graham facilitates a space for her to come into the conversation (Shakespeare, 2004). Extract 3 (line 57) is the only time across the extracts of co-remembering in which June (or any other PLWD) does not provide agreement with a candidate answer offered by Graham. June disrupts progressivity through her disalignment with Graham's candidate understanding (line 57), asserting that he has misunderstood her experience. Graham then tries again at line 58 and 60 with a new candidate answer (that June received ‘no favours’ from being the daughter of the Sunday school boss) and here confirmation in the affirmative is supplied by June (line 62).

### Using single-party memory of a shared event

Another practice used by partners of PLWD to aid progressivity is to draw on their individual memory of a shared event; resultingly the PLWD does not need to recall their own memories. The PLWD can accomplish their interactional obligations by smiling and agreeing with the account being told by their partner. Extract 4 and 5 both illustrate this practice.

*Extract 4*. Graham and June. 02:15-02:33 of 07:56 video.

*This extract continues from where Extract 2 finishes. For reference – cribbage and rummy are both card games. Polly is one of their daughters*.

5  GRA: → An’ anyway I have to play cribbage with

6     →    (0.4)

7  GRA: →  £Pol(h)ly£ huh huh

8  JUN:   Heh heh ↑heh huh

9  GRA:   She li:kes her cribbage don’t she?

10 JUN:   Yeah:.

11      (0.2)

12 GRA:    Yeah.

13     (1.4)

14 GRA: →  Now that rummy you were playin’, I’ve never played that

15    →  for yea:rs.

16   (0.8)

17 JUN:  Oh an’ nor have I. heh heh

At lines 5-7 and 14-15, Graham speaks about a recently shared experience but relates it to himself, meaning June is not required to explicitly display that she remembers (although line 9 does require a minimal confirmation from her). Graham’s turn at lines 5–7 also uses humour which allows for them both to share in laughter together. Humour is simple to respond to as a participant can laugh along and is not accountable to display why they have found something funny (Clark, 1970). The subject of the memory and humour is also something which they have shared knowledge of; their daughter’s identity as someone who likes to play cribbage.

*Extract 5.* John and Jessica. 13:10-13:23 of 22:50 video.

*Jessica and John are going on holiday soon and Jessica has told John that there’s a swimming pool at the hotel*.

9  JES: →  We could take our swimming things couldn’t we.

10 JOH:   °Yeah.°

11      ((sound of fork on the plate))

12      (0.3)

13      ((sound of fork on the plate))

14      (0.8)

15 JES:   And go for a swim.

16 JOH:     ((nods))

17      ((sound of fork on the plate))

18      (0.5)

19 JES:   I thought that’d be rather nice.

20      (0.4)

21 JES: →  We haven’t been swimming for ages.

22      (1.0)

23    [((sound of fork on the plate))

24 JOH:   [(°No. °)

25      ((sound of fork on the plate))

26 JES: →  The last time we went swimming was in Corfu.

27      (1.5 – JES turns head to JOH)

28 JOH: →  .hh huh huh huh=
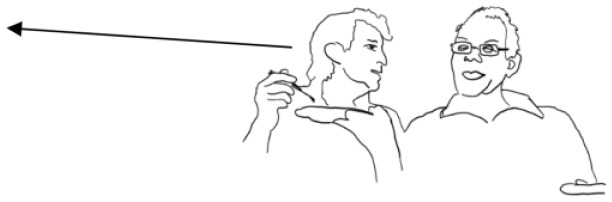

29 JES: →  =>£Do you re(h)memb(h)er.£<

30      ((clicking sound))

31 JES:   °huh hm.°

32      (.)

33 JES:   We £fou(h)nd that£ lovely deserted beach.

In Extract 5, Jessica links something happening in the future (swimming at the hotel pool) back to a memory shared by both parties. At line 9 Jessica says: ‘We could take our swimming things couldn’t we’, leading into the initiation of a memory of the last time they went swimming: ‘We haven’t been swimming for ages’ (line 21) and ‘The last time we went swimming was in Corfu’ (line 26). This is met with laughter from John (line 28), suggesting he is recalling an amusing memory. In Jessica’s next turn (line 29), her talk is interspersed with laughter particles, which latches with John’s laughter. As can be seen from the line drawing in the transcript, John’s face becomes very animated from line 28, and a wide smile stays on his face until line 40 (not included here). Jessica’s utterance of ‘Do you remember’ could display she is treating John’s laughter as displaying he remembers swimming in Corfu. Metacognitive formulations such as ‘do you remember’ happen regularly at points in interaction where the activity of remembering has become a source of interactional trouble, and even more so when one person’s account has resulted in sudden recognition in another person (Middleton and Edwards, 1990). Jessica’s ‘Do you remember’ comes after the reference (‘The last time we went swimming was in Corfu’), so the ‘Do you remember’ can be seen as a recognition check (Tao, 2001). ‘Do you remember’ in coming after the referent is similar to a tag question as it projects a next action from John (a confirmation), moving the interaction along, and favouring progressivity (Sacks et al., 1974). This displays how an individual memory of a shared event can be used to co-remember and accompanying practices which are used to aid progressivity (metacognitive formulations).

In this extract, the responses that are invited by Jessica of John are minimal, and often only agreement or assent is required. This shows how, because co-remembering is jointly constructed, it is not necessary for all parties to contribute equally to the remembering. Here, Jessica provides all the materials for the remembering through her turn design, using storytelling through discussing their future holiday and linking this back to a shared memory of when they last went swimming.

## Discussion

This article has highlighted some of the practices partners of PLWD can use during sequences of co-remembering with PLWD to keep the conversation going by maintaining progressivity. These practices are candidate answers, tag questions and single-party memory of a shared event. These findings corroborate CA research in acute hospital settings showing that healthcare professionals also tend to structure interactions with PLWD to favour progressivity, especially where the task at hand does not require intersubjective understanding (Pilnick et al., 2021). Pilnick et al. (2021) also questioned whether repair is seldom used by healthcare professionals in this context because of its potential for highlighting trouble with the PLWD’s talk and the associated impacts on their identity as a ‘competent’ speaker. We have argued in this article that by designing talk for progressivity, participants can reduce the stakes for maintaining intersubjectivity, reduce opportunities for the surfacing of interactional trouble and minimise the need for repair. As a result, PLWD and their partners can achieve collaborative co-remembering without encountering misunderstanding, mishearing or misspeaking. In designing talk for progressivity, partners can interactionally uphold the shared identity with their loved PLWD, as well as avoid challenges to PLWD’s identity as ‘competent’ speakers.

The practices identified in this article (candidate answers, tag questions and single-party memory of a shared event) create a context where assent, affiliation or agreement are acceptable responses from the PLWD, and little else is required. The practices used during sequences of co-remembering may be especially tailored to the subject of the talk (shared memories). These are memories to which all parties have equal access, in theory, although in practice, and for PLWD especially, memories may be difficult or impossible to retrieve and discuss (Alzheimer’s Society, 2021). Practices that allow for jointly significant memories to be discussed, while reducing the possibility of exposing the PLWD’s forgettings, have a two-fold benefit: not only is the PLWD interactionally included as a co-rememberer, but their agreements or assents to the partner’s tellings may also help to maintain their mutual relational identities.

However, these practices are not without risks. When the partner designs talk to favour progressivity, the conditionally relevant response is limited, and renders repair the structurally dispreferred response. For example, in line 1 of Extract 2, Graham (the partner), says to June (the PLWD): ‘It’s a bit bulky to take the Boggle when we go isn’t it’. The candidate-plus-tag-question turn design carries a structural preference for June to agree (which she does), and uphold the progressivity of the sequence, but may also limit her opportunities (and, in turn, her agency) to disagree. Of course, preference organisation is a normative, not regulative practice, and close friends or married couples can unproblematically disagree in their conversations (as has been found in other research with couples impacted by dementia: see Landmark et al., 2021). For example, in Extract 3 June disagrees with a candidate answer from Graham (line 56–57). However, this observation also points to an inherent methodological limitation of our approach to this study. We find no evidence/deviant cases in our data where PLWD express frustration or explicitly object to interactional practices that promote progressivity over intersubjectivity, although this absence of evidence may also suggest the effectiveness of such practices. It is difficult to strike an interactional balance between communicating in a way that supports agency and communicating in a way that supports progressivity. It can appear that in designing talk for progressivity, the partner sets and constrains the terms for participation. There is, however, no evidence in this dataset to support that designing talk for progressivity is treated in this way by the PLWD. The findings here suggest that partner’s design of talk for progressivity over intersubjectivity displays a method for involving the PLWD in interaction about shared memories. Research by Alsawy et al. (2020) has demonstrated how PLWD value reminiscing about shared memories, as persuasively expressed by Julie, one of their participants living with dementia, who says of reminiscing with her daughter: ‘It takes you back, it’s almost as if it was only yesterday and you know you were enjoying yourself at the time . . . If I don’t remember things she’ll remember them for me’ (Alsawy et al., 2020: 157). Although this is only one account, it illustrates the value of co-remembering and the positive impact it can have upon identity. In our dataset, it may also be that the topic (co-remembering of a shared event) and context (speaking with a close friend or spouse) of the conversation means that there is less at stake if participants contribute asymmetrically.

Another limitation of the present study is that our participants were not diverse in terms of ethnic or cultural background, although this study focuses on detailed, procedural descriptions of observed interactional practices rather than making any claims about their broader generalisability. Our participants were, however, diverse in terms of the forms of dementia they were diagnosed with and the age of the PLWD in each dyad, suggesting that these practices are common across a range of diagnoses and age groups. In future research, we could also explore interaction in co-remembering beyond verbal communication, including the wealth of other forms communication can take (e.g. in dance therapy – Nyström and Lauritzen, 2005; use of embodiment, artefacts and time – Strandoos and Antelius, 2017). There is a growing consensus in dementia studies that communicative ability is not something either present or absent in an individual, but something which is situationally and collaboratively created (Alsawy et al., 2020; Strandoos and Antelius, 2017). This is where the practices and skills used by the partner come into play in supporting interaction with the PLWD. Utilising a conversation analytic approach allows us to notice the unnoticed and reveal the skills and practices with which care partners talk with PLWD, skills which are often not explicitly recognised or valued (Handley et al., 2019; Pilnick et al., 2021). By analysing the recipient design methods that partners of PLWD use we have explicated some of the methods which can facilitate interaction with PLWD in the context of people’s own homes. This detailed analysis illustrates how it is only by interrogating the context in which interactions unfold that we can meaningfully understand the skill that the partners are displaying and what works in this specific interactional context – something generic ‘top tips’ for communication struggle to achieve (Pilnick et al., 2021).

Dementia impacts upon relationships and communication, threatening individual (Kitwood, 1997) and shared identity (Wadham et al., 2016), causing changes in communicative abilities for PLWD (Banovic et al., 2018; Nyström and Lauritzen, 2005) and creating problems in communicating effectively with PLWD for family members (Savundranayagam and Orange, 2014). In this article we have shown how interactions with others can help maintain the identity of PLWD (Cowdell, 2006; Kitwood, 1997), demonstrating the value of a relational approach for examining interactions between couples affected by dementia and how these interactions can maintain individual and shared identities. We have demonstrated how co-rememberings are an important source for conveying belonging and identity (Hydén, 2011) and answered the call for more research into the approaches used by conversation partners to support co-remembering with PLWD (Hydén, 2011). Previous research has found reminiscence therapy leads to significant increases in quality of life, and significant decreases in depression and behavioural and psychological symptoms of dementia for the PLWD who take part (Park et al., 2019). Through co-remembering past events together in the home environment, PLWD and their partners may be able to replicate some of these effects.

Our findings in this article have reaffirmed the growing consensus in dementia and disability studies that communicative ability is situationally and collaboratively created. While this research does not provide novel conversation analytical findings of its own, it does apply existing conversation analytical findings to a context that has received little exploration, contributing towards our relational understanding of interactions between informal carers and PLWD. This article shows how designing talk in a way that keeps the conversation going can help to uphold our relationships and shared memories with others and can support us to interact and maintain our individual and collective identities.

## References

[bibr1-13634593221127822] Alsawy S Tai S McEvoy P , et al. (2020) ‘It’s nice to think somebody’s listening to me instead of say “oh shut up”’. People with dementia reflect on what makes communication good and meaningful. Journal of Psychiatric and Mental Health Nursing 27(2): 151–161. 31449719 10.1111/jpm.12559

[bibr2-13634593221127822] Alzheimer’s Society (2021) Memory loss and dementia. https://www.alzheimers.org.uk/about-dementia/symptoms-and-diagnosis/symptoms/memory-loss [Accessed 3 October 2022].

[bibr3-13634593221127822] Alzheimer’s Society (2022) Dementia and language. https://www.alzheimers.org.uk/about-dementia/symptoms-and-diagnosis/symptoms/dementia-and-language#content-start [Accessed 15 May 2022].

[bibr4-13634593221127822] Antaki C (2012) Affiliative and disaffiliative candidate understandings. Discourse Studies 14(5): 531–547.

[bibr5-13634593221127822] Banovic S Zunic LJ Sinanovic O (2018) Communication difficulties as a result of dementia. Materia Socio-medica 30(3): 221–224. 30515063 10.5455/msm.2018.30.221-224 PMC6195406

[bibr6-13634593221127822] Bolden G Mandelbaum J (2017) The use of conversational co-remembering to corroborate contentious claims. Discourse Studies 19(1): 3–29.

[bibr7-13634593221127822] Clark M (1970) Humour and incongruity. Philosophy 45(171): 20–32.

[bibr8-13634593221127822] Clift R (2016) Conversation Analysis. Cambridge: Cambridge University Press.

[bibr9-13634593221127822] Cowdell F (2006) Preserving personhood in dementia research: A literature review. International Journal of Older People Nursing 1(2): 85–94. 20925734 10.1111/j.1748-3743.2006.00016.x

[bibr10-13634593221127822] Crichton J Koch T (2011) Narrative, identity and care: Joint problematisation in a study of people living with dementia. In: Candlin C Crichton J (eds) Discourses of Deficit. Basingstoke: Palgrave Macmillan, pp.101–118.

[bibr11-13634593221127822] Drew P (1997) ‘Open’ class repair initiators in response to sequential sources of troubles in conversation. Journal of Pragmatics 28: 69–101.

[bibr12-13634593221127822] Goffman E (1971 [2010]) Relations in Public. Microstudies of the Public Order. New Brunswick, Canada: Transaction Publishers.

[bibr13-13634593221127822] Goodwin C (1984) Notes on story structure and the organization of participation. In: Atkinson JM Heritage J (eds) Structures of Social Action. Cambridge: Cambridge University Press, pp.225–246.

[bibr14-13634593221127822] Goodwin MH Goodwin C (1986) Gesture and coparticipation in the activity of searching for a word. Semiotica 62: 51–75.

[bibr15-13634593221127822] Handley M Bunn F Goodman C (2019) Supporting general hospital staff to provide dementia sensitive care: A realist evaluation. International Journal of Nursing Studies 96: 61–71. 30545567 10.1016/j.ijnurstu.2018.10.004

[bibr16-13634593221127822] Harding R (2017) Duties to Care: Dementia, Relationality and Law. Cambridge: Cambridge University Press.

[bibr17-13634593221127822] Hayashi M Mori J Tagaki T (2002) Contingent achievement of co-tellership in a Japanese conversation: An analysis of talk, gaze and gesture. In: Ford CE Fox BA Thompson SA (eds) The Language of Turn and Sequence. New York, NY: Oxford University Press, pp.81–122.

[bibr18-13634593221127822] Heritage J (1984) A change-of-state token and aspects of its sequential placement. In: Atkinson JM Heritage J (eds) Structures of Social Action: Studies in Conversation Analysis. Cambridge: Cambridge University Press, pp.299–345.

[bibr19-13634593221127822] Heritage J (2007) Intersubjectivity and progressivity in person (and place) reference. In: Stivers T Enfield NJ (eds) Person Reference in Interaction: Linguistic, Cultural and Social Perspectives. Cambridge: Cambridge University Press, pp.255–280.

[bibr20-13634593221127822] Heritage J (2012) Epistemics in action: Action formation and territories of knowledge. Research on Language and Social Interaction 45(1): 1–29.

[bibr21-13634593221127822] Heritage J (2013) Epistemics in conversation. In: Stivers T Sidnell J (eds) The Handbook of Conversation Analysis. Oxford: Wiley-Blackwell, pp.370–394.

[bibr22-13634593221127822] Hydén L (2011) Narrative collaboration and scaffolding in dementia. Journal of Aging Studies 25(4): 339–347.

[bibr23-13634593221127822] Hydén L (2017) Entangled Narratives: Collaborative Storytelling and the Re-imagining of Dementia. Oxford: Oxford University Press.

[bibr24-13634593221127822] Hydén L Nilsson E (2015) Couples with dementia: Positioning the ‘we’. Dementia 14(6): 716–733. 24339120 10.1177/1471301213506923

[bibr25-13634593221127822] Jefferson G (1978) Sequential aspects of storytelling in conversation. In: Schenkein J (ed.) Studies in the Organization of Conversational Interaction. London: Academic Press, pp.219–248.

[bibr26-13634593221127822] Jefferson G (2004) Glossary of transcript symbols with an introduction. In: Lerner GH (ed.) Conversation Analysis. Amsterdam, the Netherlands: John Benjamins Publishing, pp.13–31.

[bibr27-13634593221127822] Kasper G Prior MT (2015) Analyzing storytelling in TESOL interview research. Tesol Quarterly 49(2): 226–255.

[bibr28-13634593221127822] Kemper S Lyons K Anagnopoulos C (1995) Joint storytelling by patients with Alzheimer’s disease and their spouses. Discourse Processes 20(2): 205–217.

[bibr29-13634593221127822] Kempler D (1991) Language changes in dementia of the Alzheimer type. In: Lubinski R (ed.) Dementia and Communication. Ontario, Canada: B. C. Decker, pp.98–113.

[bibr30-13634593221127822] Kindell J Keady J SAGE K , et al. (2017) Everyday conversation in dementia: A review of the literature to inform research and practice. International Journal of Language & Communication Disorders 52(4): 392–406. 27891726 10.1111/1460-6984.12298 PMC5467725

[bibr31-13634593221127822] Kitwood T (1997) The experience of dementia. Aging and Mental Health 1(1): 13–22.

[bibr32-13634593221127822] Landmark AMD Nilsson E Ekström A , et al. (2021) Couples living with dementia managing conflicting knowledge claims. Discourse Studies 23(2): 191–212.

[bibr33-13634593221127822] Middleton D Edwards D (1990) Conversational remembering: A social psychological approach. In: Middleton D Edwards D (eds) Collective Remembering. London: SAGE, pp.23–45.

[bibr34-13634593221127822] Molyneaux VJ Butchard S Simpson J , et al. (2012) The co-construction of couplehood in dementia. Dementia 11(4): 483–502.

[bibr35-13634593221127822] Nedelsky J (1989) Reconceiving autonomy: Sources, thoughts and possibilities. Yale Journal of Law and Feminism 1(7): 7–36.

[bibr36-13634593221127822] Nyström K Lauritzen S (2005). Expressive bodies: Demented persons’ communication in a dance therapy context. Health 9(3): 297–317. 15937034 10.1177/1363459305052902

[bibr37-13634593221127822] O’Brien R Beeke S Pilnick A , et al. (2020) When people living with dementia say ‘no’: Negotiating refusal in the acute hospital setting. Social Science and Medicine 263: 113188. 32823045 10.1016/j.socscimed.2020.113188

[bibr38-13634593221127822] Orange J Lubinski R Higginbotham D (1996) Conversational repair by individuals with dementia of the Alzheimer’s type. Journal of Speech and Hearing Research 39: 881–895. 8844567 10.1044/jshr.3904.881

[bibr39-13634593221127822] Park K Lee S Yang J , et al. (2019) A systematic review and meta-analysis on the effect of reminiscence therapy for people with dementia. International Psychogeriatrics 31(11): 1581–1597. 30712519 10.1017/S1041610218002168

[bibr40-13634593221127822] Peel E (2014) ‘The living death of Alzheimer’s’ versus ‘Take a walk to keep dementia at bay’: Representations of dementia in print media and carer discourse. Sociology of Health & Illness 36(6): 885–901. 24935028 10.1111/1467-9566.12122 PMC4145707

[bibr41-13634593221127822] Peel E (2015) Diagnostic communication in the memory clinic: A conversation analytic perspective. Aging and Mental Health 19(12): 1123–1130. 25647148 10.1080/13607863.2014.1003289 PMC4566896

[bibr42-13634593221127822] Peel E Harding R (2015) A right to ‘dying well’ with dementia? Capacity, ‘choice’ and relationality. Feminism & Psychology 25(1): 137–142.

[bibr43-13634593221127822] Pilnick A O’Brien R Beeke S , et al. (2021) Avoiding repair, maintaining face: Responding to hard-to-interpret talk from people living with dementia in the acute hospital. Social Science & Medicine 282: 114156. 34182355 10.1016/j.socscimed.2021.114156

[bibr44-13634593221127822] Pomerantz A (1988) Offering a candidate answer: An information seeking strategy. Communication Monographs 55(4): 360–373.

[bibr45-13634593221127822] Pomerantz A Heritage J (2012) Preference. In: Sidnell J Stivers T (eds) The Handbook of Conversation Analysis. Oxford: Wiley-Blackwell, pp.210–228.

[bibr46-13634593221127822] Rae JP Ramey M (2020) Making and taking opportunities for co-participation in an interaction between a boy with Autism Spectrum Disorder and his father. In: Wilkinson R (ed) Atypical Interaction: The Impact of Communicative Impairments. Basingstoke: Palgrave Macmillan, pp.65–92.

[bibr47-13634593221127822] Robinson L Clare L Evans K (2005) Making sense of dementia and adjusting to loss: Psychological reactions to a diagnosis of dementia in couples. Aging & Mental Health 9: 337–347. 16019290 10.1080/13607860500114555

[bibr48-13634593221127822] Sacks H (1992) Lectures on Conversation, Volumes I and II. Oxford: Wiley-Blackwell.

[bibr49-13634593221127822] Sacks H Schegloff EA Jefferson G (1974) A simplest systematics for the organization of turn taking for conversation. Language 50(4): 696–735.

[bibr50-13634593221127822] Savundranayagam MY Orange JB (2014) Matched and mismatched appraisals of the effectiveness of communication strategies by family caregivers of persons with Alzheimer’s disease. International Journal of Language & Communication Disorders 49(1): 49–59. 24372885 10.1111/1460-6984.12043

[bibr51-13634593221127822] Schegloff EA (1987) Analyzing single episodes of interaction: An exercise in conversation analysis. Social Psychology Quarterly 50(2): 101–114.

[bibr52-13634593221127822] Schegloff EA (1993) Reflections on quantification in the study of conversation. Research on Language & Social Interaction 26(1): 99–128.

[bibr53-13634593221127822] Schegloff EA (2007) Sequence Organisation in Interaction: A Primer in Conversation Analysis, Vol.1. Cambridge: Cambridge University Press.

[bibr54-13634593221127822] Schegloff EA Jefferson G Sacks H (1977) The preference for self-correction in the organization of repair. Language 53(2): 361–382

[bibr55-13634593221127822] Schegloff EA Sacks H (1973) Opening up closings. Semiotica 8(4): 289–327.

[bibr56-13634593221127822] Shakespeare P (2004) Talk, loss, and identity. Illness, Crisis & Loss 12(1): 10–22.

[bibr57-13634593221127822] Sidnell J (2010) Conversation Analysis: An Introduction. Oxford: Wiley-Blackwell.

[bibr58-13634593221127822] Small JA Gutman G Hillhouse SMB (2003) Effectiveness of communication strategies used by caregivers of persons with Alzheimer’s disease during activities of daily living. Journal of Speech, Language, and Hearing Research 46(2): 353–367. 10.1044/1092-4388(2003/028) 14700377

[bibr59-13634593221127822] Stivers T Robinson J (2006) A preference for progressivity in interaction. Language in Society 35(3): 367–392.

[bibr60-13634593221127822] Strandoos L Antelius E (2017) Interaction and common ground in dementia: Communication across linguistic and cultural diversity in a residential dementia care setting. Health 21(5): 538–554. 27895101 10.1177/1363459316677626

[bibr61-13634593221127822] Svennevig J (2012) Reformulation of questions with candidate answers. International Journal of Bilingualism 17(2): 189–204.

[bibr62-13634593221127822] Tao H (2001) Discovering the usual with corpora: The case of remember. In: Simpson RJ Swales JM (eds) Corpus Linguistics in North America. Ann Arbor, MI: University of Michigan Press, pp.116-144.

[bibr63-13634593221127822] Ten Have P (2007) Doing Conversation Analysis: A Practical Guide. London: SAGE.

[bibr64-13634593221127822] Wadham O Simpson J Rust J , et al. (2016) Couples’ shared experiences of dementia: A meta-synthesis of the impact upon relationships and couplehood. Aging & Mental Health 20(5): 463–473. 25811103 10.1080/13607863.2015.1023769

[bibr65-13634593221127822] Welland RJ Lubinski R Higginbotham DJ (2002) Discourse comprehension test performance of elders with dementia of the Alzheimer type. Journal of Speech, Language, and Hearing Research 45(6): 1175–1187. 10.1044/1092-4388(2002/095) 12546486

[bibr66-13634593221127822] Williams V Webb J Dowling S , et al. (2019) Direct and indirect ways of managing epistemic asymmetries when eliciting memories. Discourse Studies 21(2): 199–215.

[bibr67-13634593221127822] Young TJ Manthorp C Howells D , et al. (2011) Optimizing communication between medical professionals and people living with dementia. International Psychogeriatrics 23(7): 1078–1085. 21489343 10.1017/S1041610211000652

